# High-cell density two-stage self-cycling fermentation system for the enhanced production of ethanol from steam-exploded poplar hydrolysate

**DOI:** 10.1186/s40643-026-01095-4

**Published:** 2026-09-04

**Authors:** Yueh-Hao Ronny Hung, David C. Bressler, Dominic Sauvageau

**Affiliations:** 1 Department of Agricultural, Food & Nutritional Science, University of Alberta, Edmonton, T6G 2P5 Canada; 2 Department of Chemical and Materials Engineering, University of Alberta, Donadeo ICE building, 12th floor 9211 116th St NW, Edmonton, AB T6G 1H9 Canada

**Keywords:** Self-cycling fermentation, Adapted fed-batch strategy, Two-stage fermentation, Steam-exploded poplar wood hydrolysate, Lignocellulosic ethanol

## Abstract

The economics of lignocellulosic ethanol production by *Saccharomyces cerevisiae* remain a challenge due to low product yields, which can be partially addressed by improving the performance of pretreatment optimization and fermentation systems, an aspect that has received limited attention. In this study, we investigated how the combination of self-cycling fermentation (SCF) with continuous adapted feeding (controlled by evolved gas) in a two-stage system could improve bioethanol production from steam-exploded poplar hydrolysates. This system benefited from the improved productivity associated with SCF and from the high titers obtained in fed-batch operation. The system consisted of a first fed-batch SCF stage, which led to approximately two-fold improvements in cell dry weight compared to conventional batch culture and low residual glucose (< 0.5 g/L), demonstrating efficient substrate utilization by the yeast. The second high-cell density SCF stage was initiated upon reaching a metabolic threshold by adding a pulse feed in the harvested medium to further enhance ethanol titer and productivity. The patterns of glucose consumption, ethanol production, and evolved gas flow rate were all reproducible between the SCF cycles. The two-stage fermentation approach led to final ethanol titer of ~ 11% (v/v) with an overall yield of 0.453 g/g and productivity of 1.33 g/L/h (a 30% improvement over the first stage). Overall, this study presents a robust two-stage high-cell density SCF system for lignocellulosic ethanol production and highlights the feasibility and potential of implementing it in biorefinery processes.

**Supplementary Information:**

The online version contains supplementary material available at https://doi.org/10.1186/s40643-026-01095-4.

## Introduction

Lignocellulosic biomass, an abundant renewable natural resource, is considered a promising feedstock for the production of biofuel and other valuable chemicals (Patel and Shah 2021; Samantaray et al. 2026). Unlike food crops, it does not compete with food usage for fuel production (Balan 2014). Bioethanol is an important biofuel that accounts for 58% of the total liquid biofuel production according to the International Energy Agency (IEA 2023). The production steps for converting lignocellulosic biomass to bioethanol include pretreatment, hydrolysis (or saccharification), fermentation, and purification. Many studies have focused on pretreatment and hydrolysis to improve the yield and recovery efficiency of fermentable sugars for fermentation (Alvira et al. 202^2010^; Verardi et al. 2012) but fermentation itself offers opportunities to further improve microbial and processing performances. For example, the different types of fermentation – e.g. batch, fed-batch, and continuous – rely on different modes of substrate delivery, which can enhance the performance of ethanol fermentation (Hung et al. 2023; Phukoetphim et al. 2017).

Self-cycling fermentation (SCF) is a semi-continuous fermentation mode in which cycling half the volume of the fermenter upon depletion of the substrate provides the microorganisms with fresh nutrients and enhances growth (Brown 2001). SCF has found success in various applications, such as production of citric acid (Wentworth and Cooper 1996), antibiotics (Zenaitis and Cooper 1994), shikimic acid (Tan et al. 2021) and bacteriophages (Sauvageau and Cooper 2010). Of note, Wang et al. (2017) first successfully introduced the concept of SCF into ethanol fermentation by *S. cerevisiae*, demonstrating that ethanol productivity improved by 43.1% compared to batch fermentation. In a subsequent study, Wang et al. (2020) established cycling conditions for SCF in 5-L bioreactors to efficiently manage ethanol fermentation, resulting in a 37.5–75.3% increase in ethanol productivity compared to batch mode. However, due to the low substrate concentrations used, this demonstration study only led to a final ethanol titer of 2.0–2.5% (w/v), which is not sufficient for industrial production. In fact, the minimum ethanol concentration for economically viable recovery under industrial conditions is estimated at 4% (w/v) (Koppram et al. 2013; Zacchi and Axelsson 1989). In commercialized starch- and sugar-based bioethanol production, ethanol titer typically reaches above 11% (v/v) (Elliston et al. 2013). Thus, the potential implementation of SCF for ethanol production necessitates a significant increase in ethanol titer.

On the other hand, fed-batch fermentation, which involves the addition of substrates or nutrients during operation, has been shown to enhance the final product titer in many bioprocesses (Lim and Shin 2013). Chang et al. (2012) reported that final ethanol concentration increased from ~ 11 g/L to ~ 32 g/L when supplying corn cob hydrolysate during fermentation. Laopaiboon et al. (2007) improved ethanol concentration from 100 g/L to 120 g/L via fed-batch fermentation of sweet sorghum juice. Besides increased ethanol titer, fed-batch fermentation can also reduce the impact of substrate inhibition. Substrate inhibition occurs when its concentration is above a given threshold, leading to a decrease in metabolic activity (Chang et al. 2018). It is also known that overloading sugar during fermentation leads to excessive environmental osmotic pressure, resulting in reductions of yeast viability and ethanol production (Bai et al. 2008). Fed-batch fermentation is a useful approach to manage substrate delivery efficiently and circumvent some of these issues. Phukoetphim et al. (2017) investigated various feeding regimes for ethanol production from sweet sorghum juice. They found that feeding time and feeding rate influenced ethanol titer, yield, and productivity under very high gravity conditions. We previously evaluated how different adapted feeding strategies – pulsing (Hung et al. 2023) and continuous feeding (Hung et al. 2025), in which the substrate delivery rate was adjusted based on the metabolic response of *S. cerevisiae* – improved ethanol productivity.

To this end, the primary objective of the present study is to explore the performance of a two-stage integrated fermentation system for lignocellulosic ethanol production. This work represents the first demonstration of the incorporation of continuous adapted feeding (controlled by evolved gas) into SCF operation. The two-stage process enables dynamic responses to lignocellulosic environments in stage 1 and extended ethanol production to greater titers in stage 2. This was specifically designed to mitigate accumulated ethanol stresses and overcome the metabolic limitations of *S. cerevisiae* from the SCF cycle. Finally, the applicability of this integrated system to steam-exploded poplar hydrolysate was demonstrated, providing a critical proof-of-concept for achieving industrial-grade ethanol titers (~ 11% v/v) using high-cell density SCF strategies within the biorefinery industry.

## Materials and methods

### Fermentation media

#### Synthetic fermentation media

A synthetic medium, as described in Hung et al. (2023), was used to test the feasibility of the integrated fermentation system. Briefly, this medium was composed of 50 g/L glucose and 6.7 g/L yeast nitrogen base with amino acids (MilliporeSigma, Burlington, MA, USA) in 0.1 M sodium phosphate buffer (NaH_2_PO_4_$\:\bullet\:$2H_2_O/Na_2_HPO_4_$\:\bullet\:$2H_2_O, pH 6.0; Thermo Fisher Scientific, Waltham, MA, USA). For both continuous and pulsed feeding operation, the same synthetic medium was used but with glucose concentration increased to 500 g/L.

#### Lignocellulosic fermentation medium

Steam-exploded poplar (SEP), a potential lignocellulosic feedstock for the production of biofuels and other high value-added products, was prepared based on Haddis et al. (2024). The steam-exploded poplar underwent enzymatic hydrolysis as described in Beyene et al. (2017) with some modifications. 10% (w/v) steam-exploded poplar was prepared in 0.05 M sodium citrate buffer (pH 4.8) (MilliporeSigma). 20 FPU/g of cellulase cocktail NS 51,129, a non-commercial proprietary research formulation (Novozymes^®^ A/S, Bagsvaerd, Denmark), was added to the suspension. The mixture was incubated at 50 °C and 150 rpm for 24 h. After enzymatic hydrolysis, the hydrolysate was filtered through Whatman^®^ qualitative filter papers (Grade 3, diameter: 110 mm, pore size: 6 μm; MilliporeSigma) to remove solid residues. The liquid hydrolysate was then autoclaved at 121 °C/15 min to terminate the enzyme reaction. Hydrolyzed samples were taken for analyses of fermentable sugars and furfural-derived compounds (described in Sect.  2.4). The liquid hydrolysate was supplemented with 6.7 g/L yeast nitrogen base with amino acids, 0.02 g/L ergosterol (MilliporeSigma), and 0.8 g/L Tween 80 (MilliporeSigma), forming the lignocellulosic fermentation medium for subsequent experiments. Ergosterol and Tween 80 were added to the medium for the SCF system to compensate for the reduced sterol and unsaturated fatty acid synthesis in *S. cerevisiae* grown under anaerobic conditions (Wang et al. 2020).

### Yeast cultivation

Superstart™ active distillers dry yeast *Saccharomyces cerevisiae* was purchased from Lallemand Ethanol Technology (Milwaukee, WI, USA) and used in this study. The seed cultivation followed the procedure from Hung et al. (2023). In the present study, the cultivation medium for the seed culture was the same as the fermentation medium used in the bioreactor (synthetic or lignocellulosic fermentation medium).

### Fermentation configurations

#### Self-cycling fermentation with continuous adapted feeding strategy (stage 1)

Stage 1 fermentation consisted of an SCF system combined with continuous adapted feeding. It was operated in a 5-L stirred tank bioreactor (Infors-HT, Bottmingen, Switzerland). The settings of the SCF system was reported in Wang et al. (2020) with modification, and it was controlled by using a custom LabVIEW program (monitoring temperature (30 °C), agitation (600 rpm), pH, evolved gas flow rate, and cumulative evolved gas volume). Gas released from the bioreactor was monitored in real-time during the fermentation using a mass flow meter (MW-200SCCM-D/5 M, Alicat Scientific Inc., Tucson, AZ, USA). The flow rate was recorded at standard atmospheric conditions (25 °C, 1 atm) and reported as the average value over a 15-min timespan. A basic solution of 2 N NaOH (Thermo Fisher Scientific) was used to maintain pH in the first stage at a setpoint of pH 3.5 (see supplementary materials Figure S1). This setpoint was selected to ensure yeast metabolic activity and process robustness. While *S. cerevisiae* typically exhibits a physiological optimum near pH 5.0–6.0, maintaining a lower pH is common in lignocellulosic ethanol production to suppress bacterial contamination. Moreover, this setpoint aligns with the tolerance specifications (pH 3.5–6.0) of the industrial strain used in this work (Superstart™), ensuring efficient fermentation.

The first SCF cycle consisted of a batch fermentation carried out using either synthetic or lignocellulosic fermentation medium until the carbon source was depleted (identified by the evolved gas flow rate decreasing to less than 5 ccm (cubic centimeters per minute), followed by a fed-batch period in which a feed pump initiated the continuous transfer of concentrated glucose synthetic medium to the bioreactor. The feed rate was adjusted according to the parameters described in Hung et al. (2025). Thirdly, when ethanol titer reached approximately 60 g/L, the feed pump was stopped, and the cycling sequence was triggered. Ethanol production during the feeding period was monitored using evolved gas production (see supplementary materials Figure S2) and the setpoint of 60 g/L was set to avoid sugar accumulation in the system, as described in Hung et al. (2025). The cycling sequence consisted of: (1) harvest of culture broth until 1 L remained in the reactor, (2) fresh synthetic or lignocellulosic fermentation medium was added to the bioreactor until the 2-L level was reached, and (3) a new cycle was started. Nitrogen gas was purged through the bioreactor to maintain anaerobic conditions and balance the pressure of the bioreactor. The harvested medium was further transferred to shake flasks for the stage 2 pulsing fed-batch fermentation.

#### Pulsed feed second fermentation (stage 2)

200 mL of harvested fermentation medium was transferred to a sterile 500-mL shake flask, and the concentrated glucose synthetic medium was pulsed into the flask to extend ethanol fermentation and increase ethanol titer to above 11% (v/v). The stage 2 fermentation was cultured at 30 °C and 200 rpm in an orbital shaker. A S-lock was installed on the shake flask and filled with distilled water to maintain anaerobic conditions. The transition to shake flasks in stage 2 allowed for a more flexible evaluation of the final pulse feeding strategy after the primary high-cell density culture was established in the bioreactor.

### Analytical methods

Culture samples (50 mL) were collected and centrifuged at 10,100$\:\times\:$g for 10 min (Eppendorf centrifuge 5418; Eppendorf Canada Ltd., Mississauga, ON, Canada). Supernatant was taken out for analysis of sugar and ethanol contents, and the residual pellet was used for cell dry weight analysis. Sugar composition (glucose, xylose, mannose, arabinose, and galactose) of the steam-exploded poplar hydrolysate was analyzed by high-performance liquid chromatography (HPLC) (1200 series; Agilent, Santa Clara, CA, USA) equipped with an Aminex HPX-87P column (Bio–Rad Laboratory, Hercules, CA, USA) held at 85 °C, in which deionized water was used as the mobile phase with a constant flow rate of 0.3 mL/min and a refractive index detector (RID, 1100 series, Agilent), as reported by Beyene et al. (2017) with slight modifications. Potential fermentation inhibitors, furfural and 5- hydroxymethyl furfural (5-HMF), and glucose content of culture samples were analyzed by HPLC equipped with an Aminex HPX-87 H column (Bio–Rad Laboratory) using RID (Beyene et al. 2020). Ethanol titer in the fermentation medium was analyzed by gas chromatography (GC), as described in Parashar et al. (2016). Cell dry weight was measured by gravimetric analysis (Wang et al. 2020), in which experiments were performed and analyzed in at least triplicate (n$\:\ge\:$3).

## Results and discussion

### High-cell density SCF system with synthetic fermentation medium (stage 1)

**Fig. 1:**
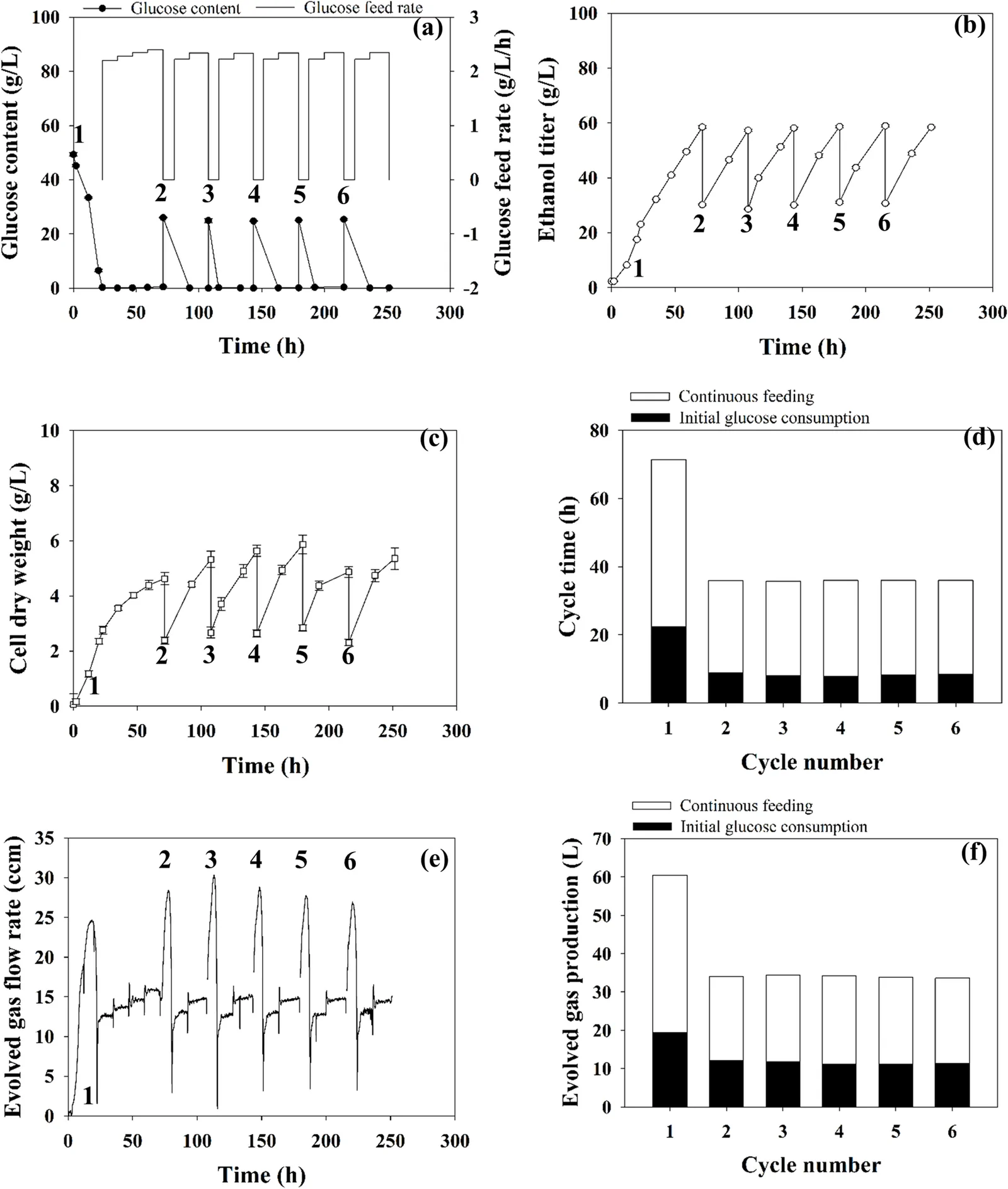
Implementing continuous adapted feeding in SCF. Glucose (**a**), ethanol (**b**), cell dry weight (**c**), cycle time (**d**), evolved gas flow rate (**e**), and evolved gas production (**f**) in synthetic fermentation medium undergoing SCF with continuous adapted feeding (stage 1). Cycle numbers are indicated at the beginning of the cycle. Means are reported from analytical triplicates with error bars representing standard deviations

In previous work, Wang et al. (2020) developed a SCF system for enhancing ethanol productivity in 5-L bioreactor, yielding approximately 2% (w/v) ethanol at the end of cycles. Basic SCF operation without nutrient supplementation was shown to be limited by initial substrate inhibition constraints. Hence, we implemented combined fed-batch and SCF operation, using a continuous adapted feeding strategy in each cycle to further increase ethanol titer and make the process more economically viable. The fed-batch strategy was selected based on improvements in ethanol productivity observed in Hung et al. (2025). Figure 1 shows trends in the fermentation parameters, including glucose concentration, ethanol concentration, cell dry weight, cycle time, and evolved gas measured over six cycles of SCF operation with continuous adapted feeding. As seen in Fig. 1a, the trend in glucose content pattern was similar for cycles 2 to 6 (these cycles are all conducted under the same feeding conditions, unlike cycle 1). These cycles were initiated at an initial glucose concentration of 25 g/L, and, upon depletion (indicated by evolved gas flow rate dropping below 5 ccm), adapted continuous feeding was initiated until the ethanol contents reached 60 g/L. As shown in our previous work (Hung et al. 2025), S. *cerevisiae* metabolic activity begins to decline once ethanol titers exceed 60 g/L during the feeding period. Hence, at this metabolic threshold, cycling was triggered. It is worth noting that the residual glucose content remained low (< 0.5 g/L) over the continuous feeding period, demonstrating that this feeding strategy did not result in substrate wastage through accumulation. Maintaining the low residual sugar in each cycle also reduced the risk of contamination, which could negatively impact ethanol yield and yeast viability (Brexó and Sant’Ana 2017). It is worth noting that no contamination was found through microscopy in our present study.

During SCF operation, ethanol titer was monitored in real time using evolved gas production as a proxy (Fig. 1e and f). The calculated ethanol contents were validated by GC analysis of samples (Fig. 1b), which showed that 60 g/L ethanol was attained at the end of cycles 2 to 6. As with glucose contents, the ethanol titer pattern was stable between cycles. Change in cell biomass is shown in Fig. 1c. In cycle 1, cell biomass reached 2.8 g/L at the end of initial batch fermentation and then increased to 4.6 g/L through the continuous feeding operation. In cycles 2 to 6, cell biomass increased from 2.6$\:\pm\:$0.2 g/L to 5.4$\:\pm\:$0.4 g/L, which was approximately a two-fold improvement within a cycle. It should be noted that the inclusion of continuous feeding to the SCF cycle led to greater biomass contents than SCF alone, as developed by Wang et al. (2020). Cycle time was 71.5 h for cycle 1, and then shortened to 36 h for cycles 2 to 6 (Fig. 1d), indicating that the same level of ethanol could be achieved in the subsequent cycles over a shorter time. When comparing between the initial batch period in SCF alone, cycle time reduced around 36% in cycle 2 to 6 as compared to cycle 1. This result is consistent with the findings of Wang et al. (2020), where cycle time stabilized to 1/3 that of cycle 1. Shortening the lag phase of the yeast and keeping its exponential growth during the subsequent cycles via cycling operation thus contributes to a reduction in cycle time for ethanol fermentation. This is supported by Feng et al. (2012), who showed that their sequential batch system eliminated the lag phase of the yeast in very high gravity conditions and enhanced annual ethanol productivity compared to the batch fermentation. Tan et al. (2021) further investigated the mechanisms increasing volumetric productivity by using transcriptomic analysis in the SCF of the engineered yeast, and the authors demonstrated that genes related to DNA replication and cell cycle were up-regulated in the early stage of SCF, leading to a higher product yield and productivity for shikimic acid production.

Under anaerobic conditions, when glucose is consumed by *S. cerevisiae*, the carbon metabolic flow is directed towards ethanol and carbon dioxide formation (Bai et al. 2008). It is important to understand the metabolic balances during ethanol fermentation. In the present work, evolved gas was monitored in real-time (Fig. 1e and f), allowing direct tracking of the performance of ethanol fermentation. The curve of evolved gas flow rate displayed a bell shape in the first part of SCF cycles (corresponding to the utilization of the initial glucose loaded) (Fig. 1e). The narrower pattern in cycles 2–6 suggests less time was required to complete a cycle compared to the initial cycle (Fig. 1d). It is worth noting that during the continuous feeding period, the evolved gas flow rate increased as glucose feed rate increased. This finding suggests a corresponding enhancement in ethanol production since it is coupled with carbon dioxide formation. This real-time monitoring also enables the establishment of automation for the SCF system, which would be expected to reduce manpower requirements (Brown 2001). The stable patterns of glucose consumption, ethanol production and evolved gas production observed over 6 consecutive cycles (equivalent to approximate 251 h of operation) also suggest the high-cell density SCF is a robust and reproducible fermentation system for ethanol production (Fig. 1f).

### Single pulsed feed fermentation with synthetic fermentation medium (stage 2)

**Fig. 2:**
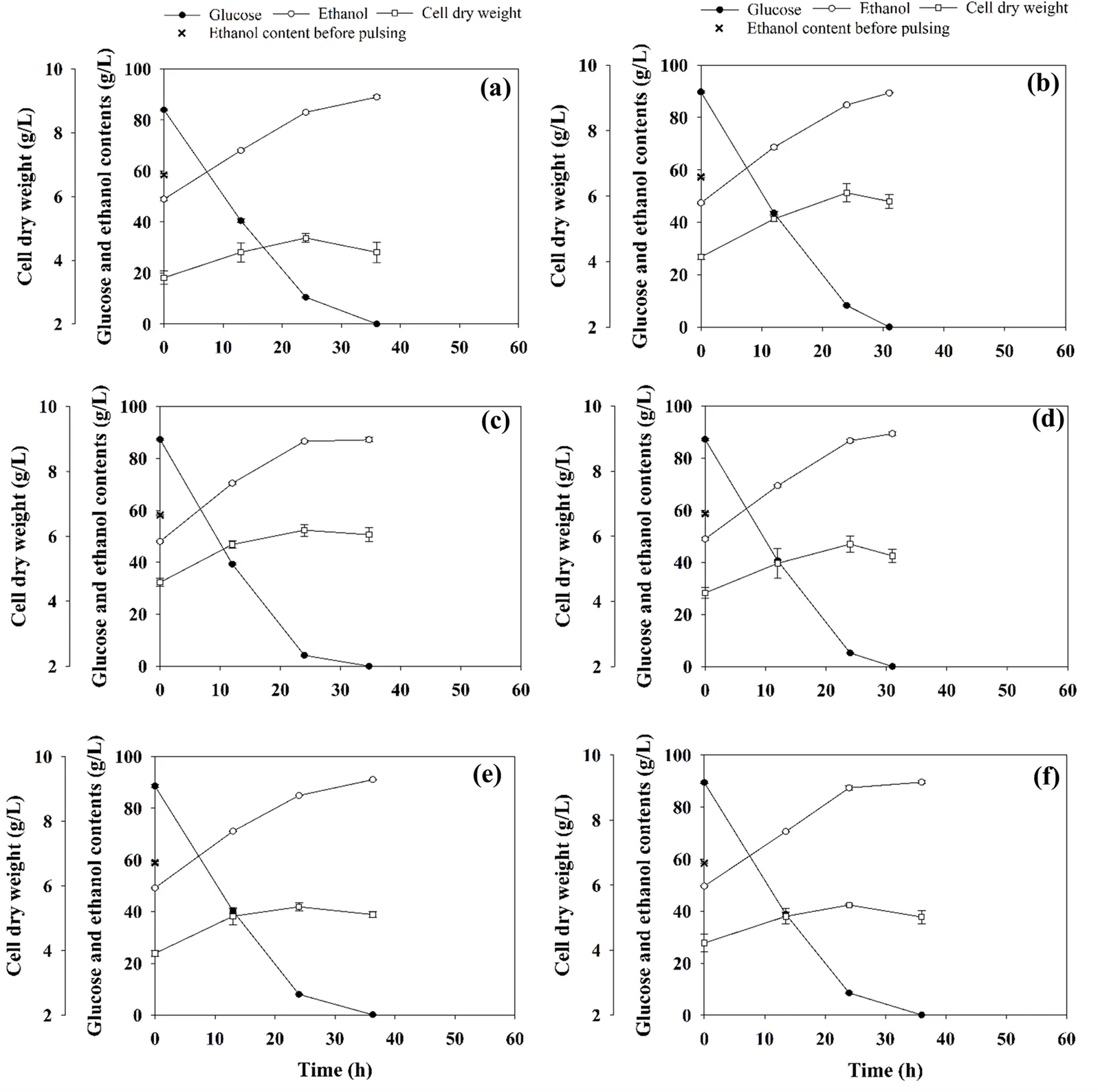
Performance of second stage in the two-stage SCF system. Glucose, ethanol, and cell dry weight in the harvested cycles 1–6 synthetic fermentation medium (**a**–**f**) undergoing stage 2. Symbol “x” shown at 0 h indicates the ethanol content in the initial harvested medium before the single pulse addition. Means are reported from analytical triplicates with error bars representing standard deviations

In the present study, a second stage was implemented in which harvested culture broth from stage 1 was transferred and supplemented with additional sugar through a single pulse addition of concentrated glucose synthetic medium. This prolonged the fermentation process to achieve higher ethanol titer. Figure 2 shows the glucose, ethanol, and cell biomass trends in stage 2 cycles 1 to 6. In each of these second stage cycles, 88$\:\pm\:$2 g/L of glucose was reduced to 7$\:\pm\:$2 g/L within 24 h, indicating that around 90% of the total glucose was consumed. In terms of ethanol titer, it should be noted that the ethanol contents reached approximately 60 g/L in stage 1; this was then diluted to ~ 49 g/L in stage 2 by adding concentrated glucose medium at 0 h (Fig. 2a–f). Additional ethanol was then rapidly produced to reach 89$\:\pm\:$1 g/L (~ 11%, v/v) at the end of fermentation. High ethanol titer can reduce the costs of downstream processing, such as ethanol recovery or waste water treatment (Viikari et al. 2012). Elliston et al. (2013) pointed out that first-generation ethanol fermentation typically reaches above 11% (v/v) to economically recover the ethanol via distillation. In our present study, ~ 11% (v/v) of ethanol was produced through the two-stage high-cell density SCF system. This finding suggests that a single pulse feed in the second stage is a feasible practice to reach a higher ethanol titer.

### Integrated two-stage system

**Fig. 3:**
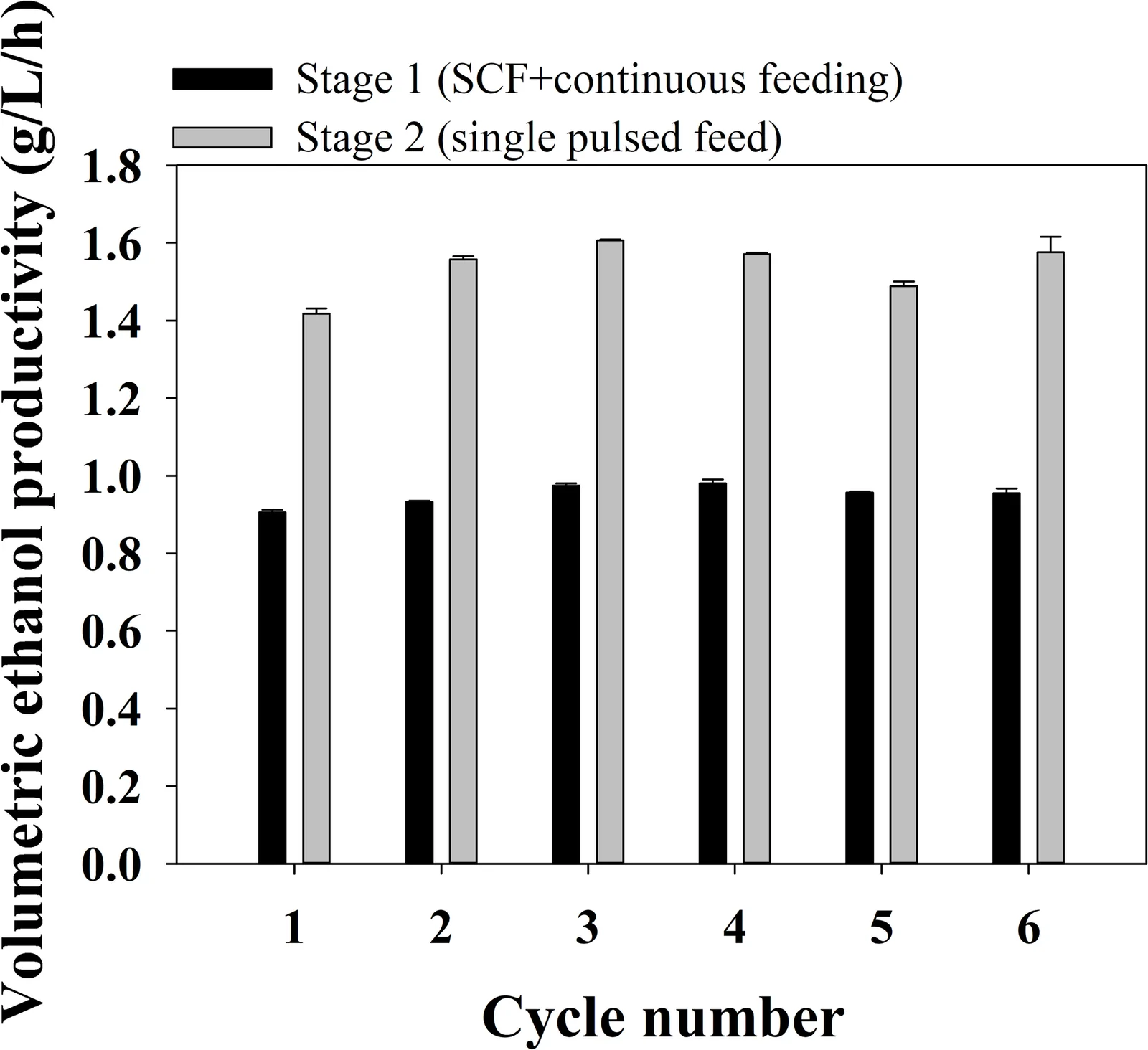
Ethanol productivity in two-stage SCF system. Volumetric ethanol productivity of the SCF with continuous adapted feeding (stage 1) and the single pulsed feed fermentation (stage 2) in synthetic fermentation medium. Means are reported from analytical triplicates with error bars representing standard deviations

It is important to note that fed-batch operation has been indicated as an effective practice for final product accumulation in many fermentation processes (Lim and Shin 2013). As demonstrated in Fig. 3, volumetric ethanol productivity in stage 2 was 60–64% higher than stage 1, showing that the single pulse approach not only elevated the final ethanol titer but also improved the ethanol productivity from the high-cell density SCF system. Intriguingly, cyclic fed-batch operation may not be favorable in a long-term operation of SCF. In a previous study, we reported that, while adapted pulsed feeding strategies could improve ethanol productivity compared to fixed pulsing strategies, yeast flocculation may occur and disrupt cell biomass homogeneity in long-term fermentation processes. Wang et al. (2020) observed yeast aggregation and flocculation in their SCF system. In the present study, yeast flocculation was observed in cycles 5 and 6. Although flocculation occurred, the feed adjustment with the continuous adapted feeding strategy was not hindered by cell aggregation as we relied on evolved gas to monitored glucose consumption rate and ethanol production during fermentation (Hung et al. 2025). As shown in Figs. 1 and 2, with a reproducible measurement of evolved gas, the continuous adapted feeding strategy could effectively support SCF.

Overall, we successfully demonstrated the feasibility of integrating a continuous fed-batch approach into a SCF system for ethanol fermentation. Table 1 highlights the fermentation performance of the high cell density SCF system (stage 1) and single pulse fermentation (stage 2) using a synthetic fermentation medium. This two-stage configuration allows for the maintenance of high metabolic efficiency and minimal residual sugar in stage 1, before transitioning to stage 2 where a single pulse feeding strategy is applied for high-titer production. The stage transition effectively mitigates the cumulative effects of cellular aging and ethanol toxicity, achieving an industrial-grade titer of ~ 90 g/L that would be operationally unsustainable in a conventional single-stage feeding process.

**Table 1:** Fermentation performance of the high-cell density SCF system (stage 1) with single pulse feed fermentation (stage 2) using synthetic fermentation medium

Cycle number	Phase	Final ethanol titer (g/L)	Ethanol yield (w/w)^#^	Volumetric ethanol productivity (g/L/h)
Cycle 1	First stage	58.5$\:\pm\:$0.4	0.431$\:\pm\:$0.005	0.906$\:\pm\:$0.007
	Second stage	89.0$\:\pm\:$0.6	0.476$\:\pm\:$0.002	1.42$\:\pm\:$0.01*
	Overall		0.454$\:\pm\:$0.002	0.968$\:\pm\:$0.006*
Cycle 2–6	First stage	58.3$\:\pm\:$0.6	0.404$\:\pm\:$0.005	0.960$\:\pm\:$0.002
	Second stage	89$\:\pm\:$1	0.459$\:\pm\:$0.009	1.56$\:\pm\:$0.04*
	Overall		0.432$\:\pm\:$0.005	1.14$\:\pm\:$0.02*

#: Ethanol yield (w/w) = (g-produced ethanol/g-consumed glucose)

*: volumetric ethanol productivity is calculated based on the ethanol production for 24 h in the second stage fermentation

In cycles 2 to 6, ethanol yield was 0.404 and 0.459 (w/w) for the first and second stage, respectively (Table 1). Considering the theoretical maximum ethanol production from glucose (0.511 g-ethanol/g-glucose) (Bai et al. 2008), the fermentation efficiency of our fermentation system reached approximately 84.5%. The slight declines in ethanol yield in cycles 2–6 compared to cycle 1 likely reflect an increased maintenance energy coefficient under the cumulative stress of recycled cell biomass and high ethanol concentrations of the later cycles, consistent with metabolic models of *S. cerevisiae* under environmental stresses (Stanley et al. 2010). However, the robust productivity seen in cycles 2–6 demonstrates the efficiency of the SCF system, where volumetric ethanol productivity shown in cycles 2 to 6 was greater than in cycle 1. This finding aligns with the report by Wang et al. (2020), who found that the subsequent SCF cycles generally had higher ethanol productivity than the first cycle. While the fermentation system proposed in the present study is not optimized, it provides proof-of-concept of the integration of different fermentation approaches to improve the overall performance of ethanol fermentation. The stable ethanol titers and higher volumetric ethanol productivity achieved in these cycles reflect a deliberate trade-off in which industrial throughput and productivity are maximized at a minor metabolic cost to conversion efficiency. In the following section, we further investigated the feasibility of applying the two-stage high-cell density SCF system to a lignocellulosic hydrolysate medium.

### Enzymatic hydrolysate from steam-exploded poplar

Table 2 shows the sugar composition of the steam-exploded poplar hydrolysate. Glucose was the primary fermentable sugar (31 g/L) in the hydrolysate, with a hydrolysis yield of 27.0%, while xylose was the second most abundant sugar (5.2 g/L), with a hydrolysis yield of 4.0%. Based on previous characterization reported in Haddis et al. (2024), the steam-exploded poplar used in this study comprised 51% cellulose (as glucan) and 13% hemicellulose (as xylan). Hence, the yields of 27% (g-glucose/g-biomass) and 4% (g-xylose/g-biomass) correspond to a recovery of 53% of the total cellulose and 31% of the total hemicellulose, respectively. While these values represent the recovery of carbohydrates under the current enzymatic conditions, it is worth noting that these recovery ratios were sufficient for the investigation of the process at hand, and that the remaining undigested solid residue holds potential for further valorization. Specifically, this undigested fraction can be processed into cellulose nanocrystals (CNCs), offering a pathway to produce high-value nano-sized particles for diverse industrial applications within a biorefinery scope (Haddis et al. 2024; Wang et al. 2021). Little arabinose, galactose, and mannose were detected in the hydrolysate with relatively low hydrolysis yield, which means they may not significantly contribute to ethanol formation compared to glucose. Furfural and 5-hydroxymethylfurfural (5-HMF), potential metabolic inhibitors of ethanol fermentation (Palmqvist and Hahn-Hägerdal 2000), were not detected in the hydrolysate (Table 2), making them unlikely to impact fermentation, leaving ethanol as the primary potential metabolic inhibitor in the system. Based on our previous findings (Hung et al. 2025) and literature (Cot et al. 2007), yeast metabolic activity and viability decline significantly when ethanol titer exceeds 60–80 g/L. Hence, the processing conditions were set to complete SCF cycles in stage 1 at approximately 60 g/L ethanol. Moreover, SCF operation mitigates the runaway accumulation of inhibitors and metabolic by-products, such as organic acids, through the replacement of 50% of the broth for each cycle. The high reproducibility of the evolved gas flow rates and fermentation kinetics across cycles 2–6 (Fig. 1) confirms that no substantial metabolic inhibition took place, keeping by-products concentrations below their inhibitory thresholds and allowing the system to maintain a functional ethanol fermentation state.

**Table 2:** Sugar composition and inhibitors in the steam-exploded poplar hydrolysate

Steam-exploded poplar hydrolysate	Concentration, g/L	Hydrolysis yield, % (g-sugar/g-biomass)
Sugars		
Glucose	31$\:\pm\:$1	27.0$\:\pm\:$0.6
Xylose	5.2$\:\pm\:$0.1	4.0$\:\pm\:$0.7
Arabinose	0.22$\:\pm\:$0.03	0.17$\:\pm\:$0.02
Galactose	0.08$\:\pm\:$0.05	0.06$\:\pm\:$0.04
Mannose	0.52$\:\pm\:$0.09	0.43$\:\pm\:$0.06
Inhibitors		
Furfural	ND	
5-hydroxylmethylfurfural (5-HMF)	ND	

ND: not detected, the concentration was less than 0.03 g/L

In addition to hexose, the hydrolysate was found to contain 5.2 g/L of xylose, which is unlikely to compete with glucose uptake as *S. cerevisiae* possesses a significantly lower affinity for xylose. This is primarily attributed to its preferential hexose transporters (e.g., Hxt2), leading to inefficient pentose uptake (Patiño et al. 2019). Moreover, the subsequent fermentation results demonstrate that glucose consumption rates remained robust throughout the SCF cycles, indicating that the presence of unutilized xylose did not interfere with the primary glycolytic pathway and/or ethanol fermentation. On the other hand, xylose utilization can be further addressed by introducing a pentose-fermenting strain (Kuhad et al. 2011), or xylose can be converted to xylitol or other value-added products for the co-production of ethanol in a market-attractive manner (Narisetty et al. 2022; Suhartini et al. 2022). In the present study, our research objective was to explore the applicability of the two-stage high-cell density SCF system using a lignocellulosic hydrolysate as feedstock. Optimization of enzymatic hydrolysis and xylose utilization are worth investigating in future research.

### High-cell density SCF system with lignocellulosic fermentation medium (stage 1)

**Fig. 4:**
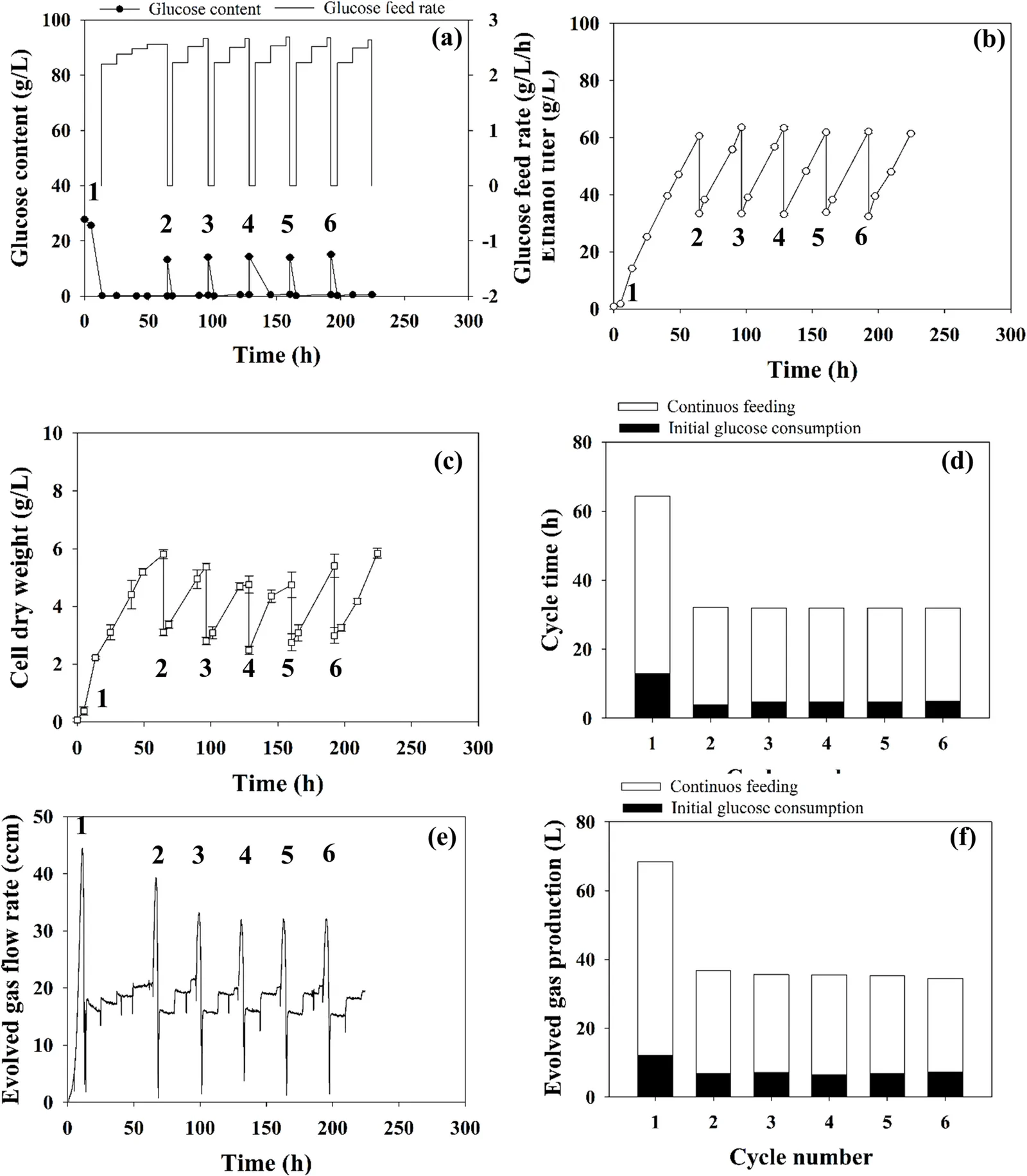
Implementing continuous adapted feeding in SCF for lignocellulosic material. Glucose (**a**), ethanol (**b**), cell dry weight (**c**), cycle time (**d**), evolved gas flow rate (**e**), and evolved gas production (**f**) in lignocellulosic fermentation medium undergoing SCF with continuous adapted feeding (stage 1). Cycle numbers are indicated at the beginning of the cycle. Means are reported from analytical triplicates with error bars representing standard deviations

In these experiments, we operated the two-stage system using the steam-exploded wood poplar hydrolysate as fermentation medium to further evaluate the effectiveness of the integrated system on a lignocellulosic feedstock. Figure 4 shows the major parameters monitored in the first stage of the high-cell density SCF system using the lignocellulosic fermentation medium. As seen in Fig. 4a–c, the patterns of glucose concentration, ethanol contents, and cell biomass were reproducible for cycles 2 to 6. Following the same operational approach, in each cycle, when the initial glucose was depleted (monitored by evolved gas flow rate), continuous adapted feeding was started to supplement substrate. It is worth noting that there was little residual glucose (< 0.5 g/L) in the fermentation medium even with a gradual increase in glucose addition during the feeding period. These findings suggest that high-cell density SCF could be effectively applied to the lignocellulosic fermentation medium. Interestingly, in terms of cell biomass, approximately 0.3 g/L of cell biomass was produced from the initial glucose consumption in cycles 2, 3, 5, and 6 (~ 4.5 h after cycling), while cell dry weight increased to ~ 2.1 g/L after implementation of the continuous adapted feeding strategy (Fig. 4c). This result also aligned with the results obtained with the synthetic medium (Fig. 1c). Here again, the reduction in SCF cycle times compared to the first cycle was substantial (64% reduction for cycles 2 to 6, Fig. 4d).

As with the synthetic medium, evolved gas flow rate was used to monitor glucose consumption, manage the continuous adapted feeding, and trigger the cycling process (Fig. 4e). Wang et al. (2021) identified that the slope of evolved gas flow rate was an effective parameter to determine the onset of stationary phase of the yeast *S. cerevisiae* growing on wood pulp hydrolysate. As seen in Fig. 4f, evolved gas production was reproducible in cycles 2 to 6, and the ethanol production in the subsequent cycles remained consistent. This observation suggests that evolved gas production is a practical monitoring parameter to estimate ethanol production from the lignocellulosic medium. Evolved gas production from continuous feeding was greater than for the initial glucose utilization, indicating most ethanol was produced in the continuous feeding stage. However, it is worth noting that should the enzymatic hydrolysis of steam-exploded poplar be further optimized, more fermentable sugars could be generated in the hydrolysate, thus improving ethanol production in the system.

### Single pulsed feed fermentation with lignocellulosic fermentation medium (stage 2)

**Fig. 5:**
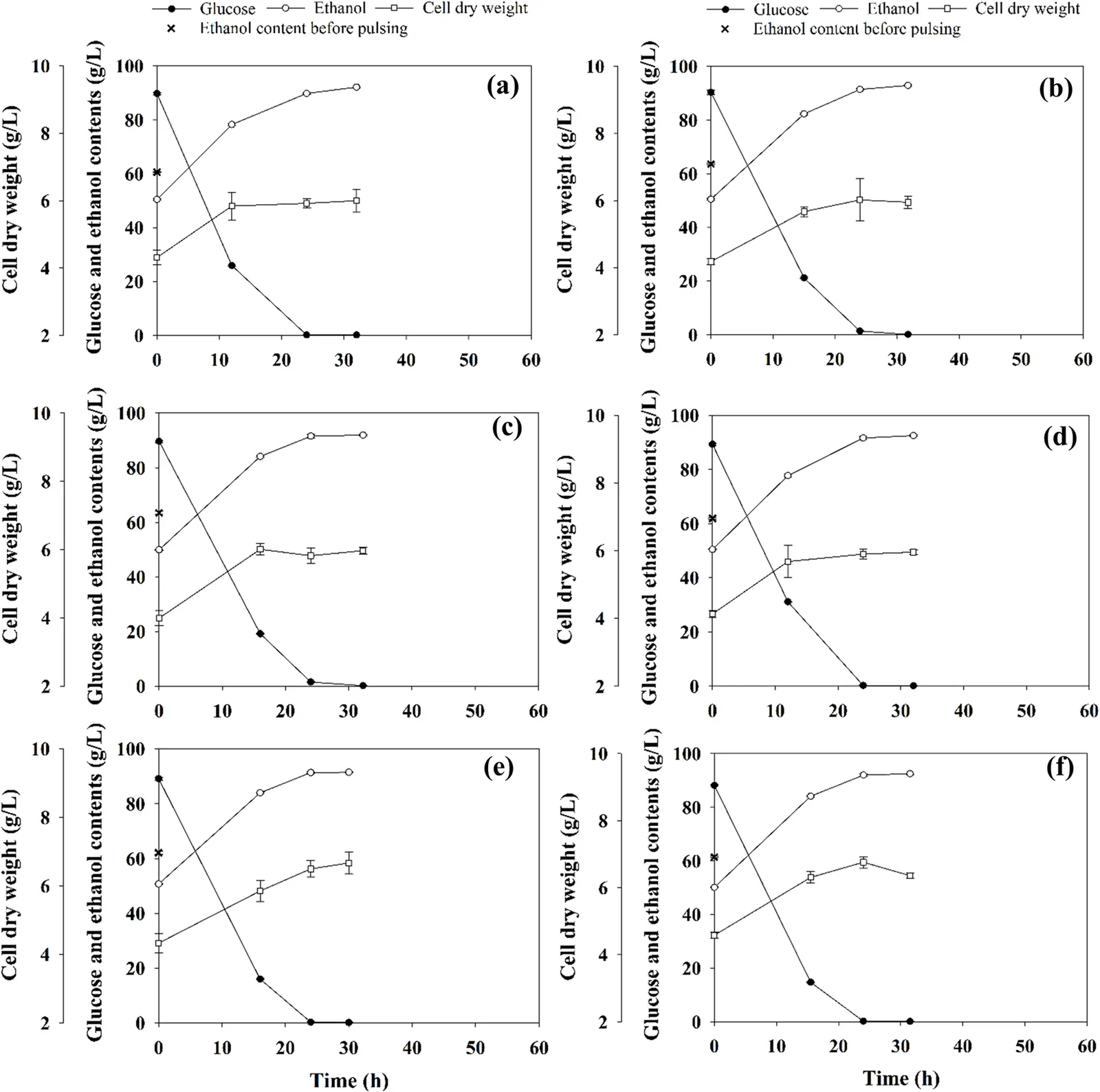
Performance of second stage in the two-stage SCF system for lignocellulosic material. Glucose, ethanol, and cell dry weight in the harvested cycles 1–6 lignocellulosic fermentation medium (**a–f**) undergoing stage 2. Symbol “x” shown at 0 h indicates the ethanol content in the initial harvested medium before the single pulse addition. Means are reported from analytical triplicates with error bars representing standard deviations

Trends in glucose, ethanol, and cell biomass in the lignocellulosic fermentation medium undergoing the single pulse feed stage 2 are summarized in Fig. 5, where Fig. 5a and f correspond to the harvested medium from cycles 1 to 6, respectively. Most glucose was consumed within 24 h in each case, and, importantly, the residual glucose was less than 0.3 g/L in Fig. 5a, d, e and f (pulsed no. 1, 4, 5, 6) at 24 h, indicating the completion of ethanol fermentation. Glucose was utilized faster in the lignocellulosic fermentation medium than in the synthetic fermentation medium of the stage 2 fermentation (Fig. 2). At 24 h, the ethanol titer reached 91.3$\:\pm\:$0.8 g/L (~ 11.6%, v/v) in all single pulse experiments (Fig. 5). This finding suggests that the integrated two-stage system can successfully be applied to lignocellulosic-based medium for high ethanol titer, a crucial indicator for the commercial potential of cellulosic ethanol production (Elliston et al. 2013). In a report by Chang et al. (2012), pulsing fed-batch fermentation was used to increase ethanol titer from corncob hydrolysate.

**Fig. 6:**
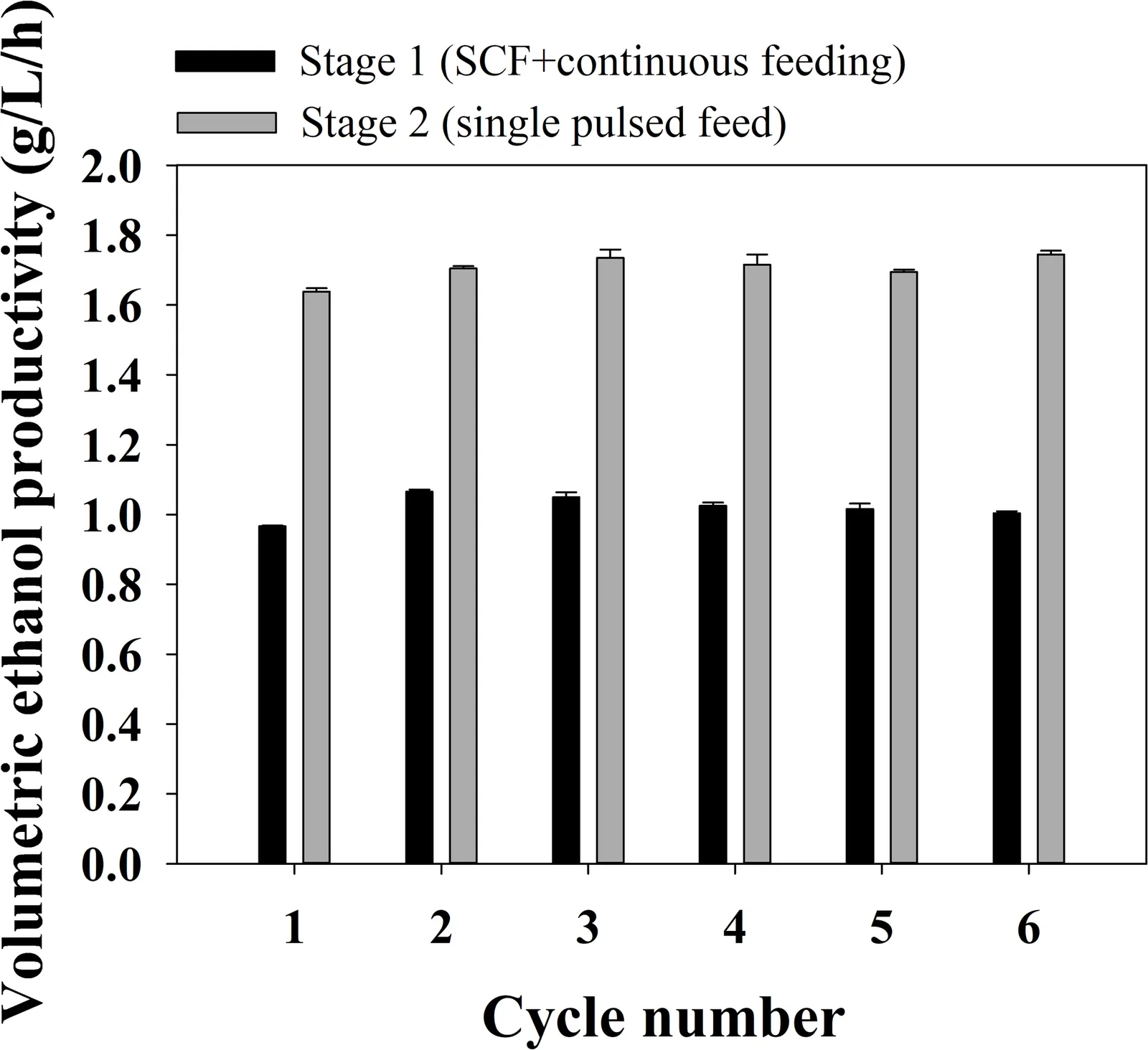
Ethanol productivity in two-stage SCF system for lignocellulosic material. Volumetric ethanol productivity of SCF with continuous adapted feeding (stage 1) and the single pulsed feed fermentation (stage 2) in lignocellulosic fermentation medium. Means are reported from analytical triplicates with error bars representing standard deviations

Figure 6 shows volumetric ethanol productivity from the high-cell density SCF system (stage 1) and the subsequent single pulse fermentation (stage 2). In addition to reaching high ethanol titer, the second stage also improved the volumetric ethanol productivity by 66$\:\pm\:$3% in cycles 1 to 6, as compared to stage 1. This is also supported by Table 3, which highlights the final ethanol titer and volumetric ethanol productivity in the lignocellulosic fermentation medium. Volumetric ethanol productivity was 1.03$\:\pm\:$0.03 g/L/h in cycles 2 to 6 of the stage 1 and was 1.33$\:\pm\:$0.02 g/L/h in cycles 2 to 6 of the overall system. Table 3 also summarizes the ethanol yield in different phases of the fermentation system, with cycle 1 reaching 0.447 (w/w) and the subsequent cycles 2 to 6 achieved 0.453 (w/w). Considering the theoretical maximum conversion of glucose (0.511 g-ethanol/g-glucose) (Bai et al. 2008), the fermentation efficiency was approximately 88% in the integrated fermentation system. It should be noted that, in the present study the concentrated glucose synthetic medium was fed to the stage 2 fermentation.

**Table 3:** Fermentation performance of the high cell density SCF system (stage 1) with single pulse feed fermentation (stage 2) in lignocellulosic fermentation medium

Cycle number	Phase	Final ethanol titer (g/L)	Ethanol yield (w/w)^#^	Volumetric ethanol productivity (g/L/h)
Cycle 1	First stage	60.6$\:\pm\:$0.1	0.430$\:\pm\:$0.001	0.966$\:\pm\:$0.002
	Second stage	92.1$\:\pm\:$0.4	0.465$\:\pm\:$0.005	1.64$\:\pm\:$0.01*
	Overall		0.447$\:\pm\:$0.003	1.14$\:\pm\:$0.01*
Cycle 2–6	First stage	63$\:\pm\:$1	0.43$\:\pm\:$0.01	1.03$\:\pm\:$0.03
	Second stage	92.3$\:\pm\:$0.6	0.470$\:\pm\:$0.008	1.72$\:\pm\:$0.02*
	Overall		0.453$\:\pm\:$0.006	1.33$\:\pm\:$0.02*

#: Ethanol yield (w/w) = (g-produced ethanol/g-consumed glucose)

*: volumetric ethanol productivity is calculated based on the ethanol production for 24 h in the second stage fermentation

To further approach the industrial practice for lignocellulosic ethanol production, the feed medium needs to be made economically, possibly utilizing bio-based feedstocks. For example, concentrated lignocellulosic hydrolysates, with mitigation or removal of inhibitors, can be more sustainable for lignocellulosic ethanol production. Similarly, sugar beet molasses, a concentrated sugar by-product from the sugar industry that has been used for ethanol production (Beigbeder et al. 2021; Ergun and Mutlu 2000), could serve as a potential replacement for synthetic glucose medium in the short-term. Furthermore, xylose utilization could lead to further optimization and improvement of the economic viability of lignocellulosic ethanol production. Xylose can be metabolized to ethanol for higher titer production by pentose-utilizing microorganisms (Kuhad et al. 2011), or it can be converted to other valuable products from the lignocellulosic fermentation medium through a biorefinery platform, such as xylitol (Kusumawati et al. 2023; Narisetty et al. 2022) and bioplastics (Nair and Verma 2025).

## Conclusions

This study successfully demonstrated a two-stage high-cell density SCF system for lignocellulosic ethanol production, providing a proof-of-concept of the integration of adapted feeding strategies into the SCF system for ethanol fermentation. Implementing the adapted feeding strategy to SCF led to approximately two-fold improvements in cell dry weight with minimal substrate wastage – residual glucose contents remained low (< 0.5 g/L) over the whole continuous feeding period. Moreover, implementing a second stage fermentation promoted the original operational fermentation pathway, ultimately achieving a high ethanol titer (~ 11%, v/v). The second stage fermentation also improved volumetric ethanol productivity by 66%, as compared to the first stage fermentation. The repeatable patterns of fermentation parameters (glucose, ethanol, and evolved gas) support the robustness and reproducibility of the integrated fermentation system. Hence, this study can act as a foundation for developing an advanced fermentation system for lignocellulosic ethanol production, enhancing its feasibility for commercialization.

## Supplementary Information

Supplementary Material 1.

Supplementary Material 2.

## Data Availability

The authors confirm that the data supporting the findings of this study are available within the article and/or its supplementary materials on request.
