# Supplementary material for: High-cell density two-stage self-cycling fermentation system for the enhanced production of ethanol from steam-exploded poplar hydrolysate
Source: Bioresour Bioprocess. 2026 Sep 4;13(1):130. doi: 10.1186/s40643-026-01095-4 (PMC13545163; doi:10.1186/s40643-026-01095-4)
Supplement: Supplementary file 1 [file 40643_2026_1095_MOESM1_ESM.docx]

**Appendix: Supplementary Materials**

**Ethanol production from steam-exploded poplar hydrolysate through a two-stage high-cell density self-cycling fermentation system**

Number of Supplementary Figures: 2

**(a)**

**1**

**2**

**3**

**4**

**5**

**6**

**(b)**

**1**

**2**

**3**

**4**

**5**

**6**

**Figure S1.** pH in the SCF with continuous adapted feeding (stage 1) from synthetic fermentation medium (a) and lignocellulosic fermentation medium (b). Cycle numbers are indicated at the beginning of the cycle.

| Evolved gas produced (mL/L) | Ethanol produced (g/L) |
| --- | --- |
| 4788.75 | 9.26531 |
| 2553.1 | 4.24534 |
| 2555.85 | 6.06005 |
| 5476.95 | 7.76227 |
| 2276.05 | 3.46573 |
| 3264.55 | 5.58483 |
| 5860.3 | 10.0082 |
| 2881.4 | 4.14741 |
| 2839.95 | 2.61971 |
| 5249.3 | 9.10624 |
| 2329.5 | 2.5722 |
| 2108.95 | 0.987102 |
| 3060.1 | 5.13348 |
| 4813.1 | 9.17188 |
| 2626.85 | 4.27084 |
| 2658.7 | 3.2426 |
| 2863 | 2.59758 |
| 2881.45 | 4.24856 |
| 5979.55 | 10.3162 |
| 2927 | 3.60527 |
| 2843.4 | 3.6091 |
| 5196.5 | 9.09519 |
| 2303.45 | 2.83811 |
| 3426.55 | 3.74783 |
| 4609.8 | 8.37236 |
| 2465.7 | 4.23363 |
| 2464.05 | 4.20578 |
| 5192.45 | 9.93214 |
| 2676.5 | 4.89035 |
| 2692.7 | 3.7593 |
| 5669.8 | 9.89203 |
| 2809.25 | 4.16138 |
| 2792.45 | 3.36995 |
| 5211.6 | 9.8986 |
| 2358.4 | 2.21376 |
| 3528.4 | 4.23223 |

**Figure S2.** The correlation between evolved gas produced (mL/L) and ethanol produced (g/L) per reactor volume in ethanol fermentation of *S. cerevisiae*.
